# Immunological modulation of estrogen during sepsis

**DOI:** 10.1186/cc11714

**Published:** 2012-11-14

**Authors:** AM Stabile, ME Batalhão, EC Carnio

**Affiliations:** 1 University of Sao Paulo, College of Nursing at Ribeirão Preto, Ribeirão Preto, Brazil

## Background

Sepsis and its common complication septic shock, are generally induced by the action of lipopolysaccharide (LPS) and is characterized by peripheral arteriolar vasodilatation which results in hypotension and inadequate tissue perfusion. Nitric oxide (NO) is a free radical gas, produced by the immune system in response to an immunological stimulus and is related to the pathogenesis of sepsis due to its vasodilator and cytotoxic actions. One significant finding in clinics is that man and woman respond differently to sepsis, with better prognosis related to women [1].

## Objective

This study was designed to investigate the role of estrogen on immune response in an experimental model of septic shock.

## Methods

In the first set of experiments male and female (ovariectomized and sham surgery) rats were injected intraperitoneally (IP) for three consecutive days with estradiol cypionate (ECP), 40 μg/kg or vehicle. In the third day, after ECP injection, rats receive IP injection of 10 mg/kg of bacterial LPS or saline solution. Plasma was collected 4 and 6 hours after LPS. In the second set of experiments macrophage culture was performed from peritoneal wash of male and female rats. Culture was stimulated with β-estradiol (10^-9^M) and LPS 1 μg/ml and medium collected 12 and 24 hours after stimulation for NO measure.

## Results

*In vivo *experiments showed that administration of LPS increased NO plasma concentration in males and females. ECP pre-treatment decreased NO concentration in sham females in the two studied periods (4 and 6 hours); conversely, increased nitrate levels in ovariectomized and had no effect in males. Results of *in vitro *experiments showed that macrophages from males produced more NO than the ones from female, either in basal conditions or after LPS stimulation; however, treatment of the culture with β-estradiol, significantly increased the NO production in macrophages from male but had no effect in female rats.

## Conclusion

Our results indicate that estradiol may have pro or anti-inflammatory actions depending on the gender; however, estradiol in female seems to be protective, since it decreased NO plasma concentration. These results may explain in part the better outcomes of woman during sepsis.
